# Dietary diversity, feeding selectivity, and responses to fruit scarcity of two sympatric Bornean primates (*Hylobates albibarbis* and *Presbytis rubicunda rubida*)

**DOI:** 10.1371/journal.pone.0173369

**Published:** 2017-03-09

**Authors:** Dena J. Clink, Christopher Dillis, Katie L. Feilen, Lydia Beaudrot, Andrew J. Marshall

**Affiliations:** 1 Department of Anthropology, University of California-Davis, Davis, California, United States of America; 2 Graduate Group in Ecology, University of California-Davis, Davis, California, United States of America; 3 Conservation Department, Disney’s Animals, Science and Environment, Lake Buena Vista, Florida, United States of America; 4 Department of Ecology and Evolutionary Biology and Michigan Society of Fellows, University of Michigan, Ann Arbor, Michigan, United States of America; 5 Department of Anthropology, Program in the Environment, and School of Natural Resources and Environment, University of Michigan, Ann Arbor, Michigan, United States of America; University of Illinois at Urbana-Champaign, UNITED STATES

## Abstract

Effectively characterizing primate diets is fundamental to understanding primate behavior, ecology and morphology. Examining temporal variation in a species’ diet, as well as comparing the responses of different species to variation in resource availability, can enhance understanding of the evolution of morphology and socioecology. In this study, we use feeding data collected over five years to describe the diets of two sympatric Southeast Asian primate species of similar body size: white-bearded gibbons (*Hylobates albibarbis*) and red leaf monkeys (*Presbytis rubicunda rubida*), in Gunung Palung National Park, West Kalimantan, Indonesia. Long-term data sets are especially important for characterizing primate diets in Southeast Asia, where the forests exhibit supra-annual mast fruiting events. We found that gibbons were mainly frugivorous, with fruit and figs comprising 70% of their 145 independent feeding observations, whereas leaf monkeys ate a substantial amount of seeds (26%), fruits and figs (26.5%) and leaves (30%, n = 219 independent feeding observations). Leaf monkeys consumed a higher number of plant genera, and this was due mostly to the non-frugivorous portion of their diet. To investigate resource selection by these primates we utilized two different approaches: the Manly Selectivity Ratio, which did not take into account temporal variation of resource availability, and a model selection framework which did incorporate temporal variation. Both species selected figs (*Ficus*) more than predicted based on their availability under the Manly Selectivity Ratio. Model selection allowed us to determine how these primates alter the proportion of leaves, flowers, seeds, figs and fruit in their diets in response to variation in fruit availability. When fruits were scarce, both gibbons and leaf monkeys incorporated more leaves and figs into their diets, indicating that these two food classes are fallback foods for these primates. We discuss how different measures of resource selection can provide seemingly contradictory results, and emphasize the importance of long term studies that combine independent feeding observations with rigorous assessment of temporal variation in resource availability when modelling feeding selectivity.

## Introduction

A thorough investigation of primate diets, and how primates alter their diets in response to variation in food availability, is fundamental for understanding primate behavior, ecology and morphology [1–9]. Periods of resource scarcity may have particularly important impacts on primate fitness because during these times feeding competition can be intense and food quality poor [10]. While such periods can have disproportionate impacts on primate feeding adaptations and sociality, they occur infrequently in some environments [9,11–13]. Long-term observations of primate feeding behavior and concurrent assessment of plant food availability are therefore necessary to sample across the full range of variation within the diet and to encompass periods of high and low resource availability [14–16].

The need for long-term data sets is particularly acute in Southeast Asia because most forest types there exhibit dramatic, supra-annual fluctuations in fruit production that exceed the magnitude of variation in food availability characteristic of other tropical forests [17–20]. Mast fruiting events are periods of super-abundance of resources, and are characteristically followed by periods of extreme food scarcity [14,19]. These phenological cycles are linked to EL Niño Southern Oscillation events [18,21] and consequently occur at irregular intervals that are unpredictable from the perspective of vertebrate frugivores. Due to the hyper-variability in food availability in the Dipterocarp forests of SE Asia, dietary changes in response to food availability can be dramatic, with some primate species incurring negative energy balance during periods of low resource availability [22]. Studying the responses of frugivores to these fluctuations in food availability—especially the responses of multiple taxa that differ in their dietary adaptations, life histories, and feeding strategies [23,24]–can shed light on the evolution of primate feeding adaptations.

A useful way to understand dietary responses to fluctuations in food availability is to categorize dietary items based on their use and availability, and in particular to distinguish between preferred and fallback foods [9,25–28]. Preferred foods are generally high-quality foods that are easy to process and are eaten more often than would be predicted based on their availability [29]. Foods that are consumed more during periods when preferred foods are scarce are termed fallback foods [9]. Comparative studies of primate diets are particularly informative for understanding how responses to resource availability drive evolutionary processes [30–32]. For example, the African grey-cheeked mangabey (*Lophocebus albigena*) has a relatively high degree of dietary overlap with the sympatric red-tail guenon (*Cercopithecus ascanius*). *L*. *albigena* possesses much harder tooth enamel than *C*. *ascanius*, some of the hardest tooth enamel found in extant primates. The foods that *L*. *albigena* consumes during times of resource scarcity (i.e., their fallback foods: bark and seeds) are thicker and harder to process than foods eaten by *C*. *ascanius*, and the difference in tooth enamel thickness between the two species can be explained by the foods they consume when resources are scarce [8]. Comparative studies can also be useful for understanding how resource availability influences primate population biology. For example, the population density of white-bearded gibbons (*Hylobates albibarbis*) is limited by the availability of their fallback foods [9,14], whereas red leaf monkey (*Presbytis rubicunda*) population density is limited by the availability of high quality, preferred foods [24]; these differences may be due to differences in the life histories of the two species [25].

Gibbons and leaf monkeys provide an excellent comparison for investigating the effects of resource variability on primate ecology because they are similar in body size, but have different social systems, life histories and diets [24]. Gibbons generally live in male-female pairs and have relatively slow life histories (e.g., long inter-birth intervals and long lifespans), whereas leaf monkeys live in single-male, multi-female groups and have relatively fast life histories [25]. Gibbons and leaf monkeys are classified as frugivores and folivores/gramnivores, respectively. Gibbons are generally considered ripe-fruit specialists and possess few morphological adaptations to process low-quality foods [33], whereas leaf monkeys, like all colobine monkeys, have morphological adaptations such as complex, multi-chambered stomachs, thin tooth enamel and high shearing cusps that facilitate the consumption of leaves [34,35].

In this study, we conduct a dietary analysis of two sympatric primate species, red leaf monkeys (*P*. *rubicunda*, hereafter referred to as leaf monkeys), and white-bearded gibbons (*H*. *albibarbis*, hereafter referred to as gibbons) in Gunung Palung National Park, Indonesia using plant phenology data and primate feeding observations collected over 66 months. We examine the feeding ecology of sympatric populations of gibbons and leaf monkeys to: 1) characterize and compare gibbon and leaf monkey diets, identify the genera consumed and their importance, the relative contribution of different plant parts to overall diets, and overall dietary richness, diversity and overlap; 2) analyze feeding selectivity for each primate species; and 3) assess how these primates respond to temporal variation in fruit availability. Specifically, we make the following predictions: compared to gibbons, leaf monkeys will have higher dietary richness and diversity; prefer more genera, and avoid fewer genera; and show shifts in types of plant parts consumed in response to variation in overall fruit availability. We make these predictions based on evidence that leaf monkeys have morphological and physiological adaptations to process a wider variety of foods than gibbons [36–38].

## Materials and methods

### Field site and study subject

We conducted this study at the Cabang Panti Research Station (CPRS) in Gunung Palung National Park, West Kalimantan, Indonesia (1°13° S, 110°7° E) from September 2007 through February 2013. At CPRS, mean gibbon group sizes are 4.32 individuals (SD = 0.89, range 2–6, N = 33 groups) and mean home range size is 43 ha (SD = 5.4); mean leaf monkey group sizes are 5.77 individuals (SD = 2.6, range 2–11, N = 13 groups) and with 90 ha (SD = 11.4) mean home range size [24,39]. There are seven floristically distinct forest types at Gunung Palung National Park [20], but for the present analyses we focused on the five forest types that exhibit mast fruiting (freshwater swamp, alluvial bench, lowland sandstone, lowland granite, and upland granite) as the non-masting forest types (montane and peat swamp) have dramatically different phenological patterns and plant species composition [14,18,20,23]. We operationally define mast fruiting events as periods where there was at least a three-fold increase in fruiting stems above the mean proportion of stems fruiting in all other months. We recorded daily maximum and minimum temperature and rainfall at the field station at CPRS (elevation approximately 15 m asl).

### Feeding observations

Each month, AJM, field managers, or trained Indonesian field assistants walked two replicate census routes (averaging 3.5 km in length) in each of the seven forest types found at CPRS and collected data on gibbon and leaf monkey feeding behavior. Inter-observer reliability was ensured through extensive training, periodic checks of distance measures, and regular quizzes to assess the accuracy of plant and vertebrate species identifications. Observers were randomly rotated across habitat types and census routes, and average encounter rates and detection distances are highly concordant between observers [39]. Standard line-transect methods allowed for the collection of statistically independent feeding observations and avoided the potential for pseudo-replication that may occur when multiple feeding observations are collected from the same group on the same day. We systematically walked fourteen, spatially segregated line transects, at a consistent speed between 0530 and 1200 hrs (for details see [14,40]). For any group or individual encountered while feeding, we recorded the first item consumed by the first individual seen [40,41]. We collected feeding data on all age and sex classes, thus adults and juveniles of both sexes were included in our analyses. Because data were collected across multiple forest types and many groups, the results reflect the diet for the population, rather than potentially idiosyncratic observations of a single group. Following collection of feeding data, observations along the vertebrate census route continued so that multiple feeding observations were not made from the same group on the same day.

We collected additional feeding data during targeted focal observations of gibbons and leaf monkeys. We selected target groups at random from among the known groups at the site (N_HYLOBATES_ = 20–28 groups, N_PRESBYTIS_ = 8–14 groups during the research period located across the five masting forest types examined in this study). After contacting the target primate group (normally in the morning between 6:00 and 9:00, although some observations were made later in the day), we randomly selected a focal individual of any age-sex class (except nursing infants) and followed until it began feeding. Data collection on focal follows continued for 30 minutes, at which point a new focal individual was randomly chosen. We did not record a new feeding observation from the focal animal until it had travelled to a different tree or liana to ensure that multiple feeding observations were not recorded from the same individual plant.

We collected the following data for each primate group encountered on transect routes and during focal follows. For the plant fed upon by the first primate individual sighted, we recorded the identification of the plant eaten (to the lowest taxonomic level possible), location (using a GPS unit and/or a detailed address based on the trail system), size (dbh, diameter at breast height), and growth form (i.e., tree, liana) of the plant; the part being eaten (e.g., fruit pulp, seeds, young leaves); the maturity stage, if applicable (e.g., immature, ripe); the number of animals feeding; and an estimate of the total crop size [14,39]. We gathered one feeding observation every 3.6 days, on average (range 0–68, SD = 6.3). In previous analyses, we found there were no significant differences in the use of plant genera collected during line transect surveys or focal follows [41], therefore we lumped feeding observations together to increase sample size.

### Assessing plant phenology

To assess spatial and temporal variation in food availability, we monitored the reproductive behavior of tree and liana stems located in fifty 0.1 or 0.2 ha botanical plots (placed in a random, stratified manner, 10 plots and 1.5 ha per forest type; 4,739 tagged stems, see [24,41]). Each month all stems in every plot were carefully examined with binoculars and assigned to one of six reproductive states (reproductively inactive, or bearing flower buds, flowers, immature, mature, or ripe fruits). Determination of fruit ripeness stages was based on changes in size, color, and texture, using categories developed over the last 30 years for each plant taxon [20,25]. Mature fruits are full-sized fruits that are unripe but have seeds that are fully developed and hardened; ripe fruits are the final development stage prior to fruit fall, usually signaled by a change in color or softness [42].

Bornean forests contain some of the highest levels of vascular plant diversity in the world [43], and although many plant stems in the plots were identified to species, the inclusion of stems identified only to genus meant that all analyses were done at this higher taxonomic level. Previous work on a variety of taxa has shown that lower taxonomic resolution is appropriate when identification to the species level is not possible or feasible [44–46].

### Dietary composition

We used rarefied species accumulation curves to assess the dietary richness (i.e., taxonomic breadth) of gibbon and leaf monkey diets, both overall and for the frugivorous portion (excluding figs) of the diet. We report dietary richness as the observed cumulative number of plant genera consumed given the number of months in which feeding observations were recorded for each primate species. We constructed diet richness curves using the “specaccum” function from the “vegan” package [47] in R 3.0.0 statistical software [48].

We described diet breadth of gibbons and leaf monkeys both in terms of the number plant taxa eaten and the relative proportion of feeding observations on each of five food classes (leaves, flowers, seeds, fruits, and figs). We exclude the synconia of *Ficus* (Moraceae) from the category of fruits and treat them separately because their reproductive parts are not true fruits, they are important foods for many vertebrates [25,49,50], and they have unusual phenological behavior rendering them qualitatively distinct from other fruiting plants considered herein [18,41,51].

### Dietary overlap

We calculated dietary overlap measures that incorporated the number of shared items in the diet and their relative importance [40]. Our dietary overlap index ranges from 0 to 1, with 0 indicating no overlap and 1 indicating complete overlap. To calculate the dietary overlap index, we tallied the number of feeding observations on each genus for both gibbons and leaf monkeys, including multiple feeding observations on the same food item when applicable. We then compared the overlap of items eaten by both primates to the total number of items eaten by each primate. Thus, from the perspective of consumer A, the equation is:

A∩Bn/An

and from the perspective of consumer B:

A∩Bn/Bn

where *A* ∩ *Bn* is the number of food items shared by the two consumers, and *An*, *Bn* are the number of food items in each of the respective consumer’s diets [40]. As gibbons and leaf monkeys had different total numbers of feeding observations, and varying numbers of feeding observations on the same genus (i.e., different genera were of varying importance), this resulted in different values of dietary overlap for each species, with each value representing dietary overlap from the focal consumer’s perspective. Dietary overlap was calculated for the overall diet and for each food class (fruits, seeds, flowers, leaves and figs).

### Dietary selectivity

Our study focused on the fruit portion of the diets, because fruits are expected to be the most limiting class of resources on which these species feed [14,24,40]. Forest productivity was defined for gibbons as the proportion of stems bearing fruit that were mature or ripe; for leaf monkeys stems bearing immature and mature fruits were also included, reflecting the fact that gibbons avoid unripe fruits whereas leaf monkeys consume them frequently and tend to avoid ripe fruits because foods rich in starch or sugar can disrupt the forestomach pH and cause acidosis [38]. Despite this fact, we included ripe fruits in the estimate of food availability for leaf monkeys as our phenology categories were based on the most advanced stage, meaning that trees with one ripe fruit were scored as ripe, even if most of the fruits on the tree were still mature [14,42], so plants scored as ripe often still contained some food for leaf monkeys. We followed the Design I Protocol for calculating selectivity ratios, the appropriate approach when animals are not individually identified, the availability of a given resource is known, and resource use is sampled randomly [46].

We calculated Manly Selectivity Ratios (MSRs; selection ratio: genus use/genus availability) using the “widesI” function in the “adehabitat” package in R [52]. Genus use is simply the total number of independent feeding observations recorded on a genus, *i*. We followed the general convention for MSRs and calculated availability as the total number of stems of genus *i* that were observed to fruit during the study period. We calculated selection ratios for each genus that was observed to have been fed upon at least once by either gibbons or leaf monkeys. We report MSRs that are standardized so that they add to 1; these values can be interpreted as the probability that for a selection event the primate would choose the genus of interest over other available genera [53]. Values close to zero indicate “avoidance”, meaning the genus was eaten less than would be expected based on its availability. Thus, in the MSR context, the term “avoidance” of an item does not necessarily indicate the item is never consumed (i.e., it does not mean that it is a non-food item). Large values indicate “preference” wherein genera were selected more than predicted based on availability.

We conducted a chi-squared test of the null hypothesis that the animals were randomly feeding (i.e., selecting resources in proportion to their availability). The chi-squared test was significant for both animals, so we computed 95% confidence intervals for proportions of used and available resources. If there are fewer than five resource units per category (or genera in this case), the corresponding confidence intervals should be interpreted with caution [53]. Relatively few of the genera in our study had five or more feeding observations (5 genera for gibbons and 8 genera for leaf monkeys), so we caution against strong interpretations based on the calculated confidence intervals of the selectivity values.

### Dietary composition as a function of fruit availability

We calculated the proportion of the diet comprising each of the five food classes in three-month blocks. We combined data into three-month periods in order to increase sample size per period and thereby improve the reliability of estimates [41]. We then compared the number of feeding observations for each food class (figs, flowers, fruit, leaves and seeds) in each three-month block to the corresponding average fruit availability values during the same period. Availability values were calculated as the proportion of the overall stems in the forest that were bearing fruit. Availability differed for gibbons and leaf monkeys because immature fruits were included in calculations for the latter but not the former (see above).

We fit linear models using ordinary least squares regression that predicted use (number of feeding observations on a given food class) based on the following predictors, calculated for a three-month block: *fruit availability* (proportion of stems in plots that were fruiting), *seed availability* (proportion of stems in plots with seeds), *flower availability* (proportion of stems in plots that were flowering), and *fig availability* (proportion of stems of the genus *Ficus* that were fruiting). We also included the following environmental predictors: *minimum temperature* (the mean minimum temperature for a given three-month block), *maximum temperature* (the mean maximum temperature for a given three-month block), and *rainfall* (average daily rainfall, in centimeters, for a given three-month block). We then compared each of these models and a null intercept model using biased-corrected Akaike's Information Criterion (AICc) using the “AICctab” function in the R package “bbmle” [48]. We obtained estimates of the 95% confidence bands for the regressions by simulation using the R package “rethinking” [54]. Due to random sampling variation, some three-month blocks had observations of leaf monkeys but none for gibbons, so we fit the models using 15 three-month blocks for gibbons and 20 three-month blocks for leaf monkeys.

### Ethics statement

This research complied with all applicable laws of the Republic of Indonesia and the United States of America. Per regulations of the Institutional Animal Care and Use Committee at the University of California-Davis, as our research entailed solely non-invasive observation of wild animals, no formal IACUC review was required. Permission to conduct research at Gunung Palung National Park was kindly granted by the Indonesian Institute of Sciences (LIPI), the State Ministry of Research, Technology, and Higher Education (MENRISTEKDIKTI), the Directorate General for Nature Conservation (PHKA) and the Gunung Palung National Park Bureau (BTNGP).

## Results

We collected 145 feeding observations from gibbons and 219 feeding observations from leaf monkeys (gibbon feeding data in S1 Table and leaf monkey feeding data in S2 Table). Mean monthly survey effort across the five forest types sampled was 61.45 km/month (SD = 2.73 km). Approximately 41% of feeding observations were recorded on census routes (N_GIBBONS_ = 102 observations or 46% of total gibbon observations, N_LEAF MONKEYS_ = 120 observations or 38% of total leaf monkey observations), and the remainder were made during focal follows. Most feeding observations (88% of all observations) were made in the morning. There was no systematic bias towards males or females in either species, nor any difference in the proportion between them. Our observations were mostly of adults (~75%), but the proportion of adults and subadults did not differ between species.

**Fig 1:**
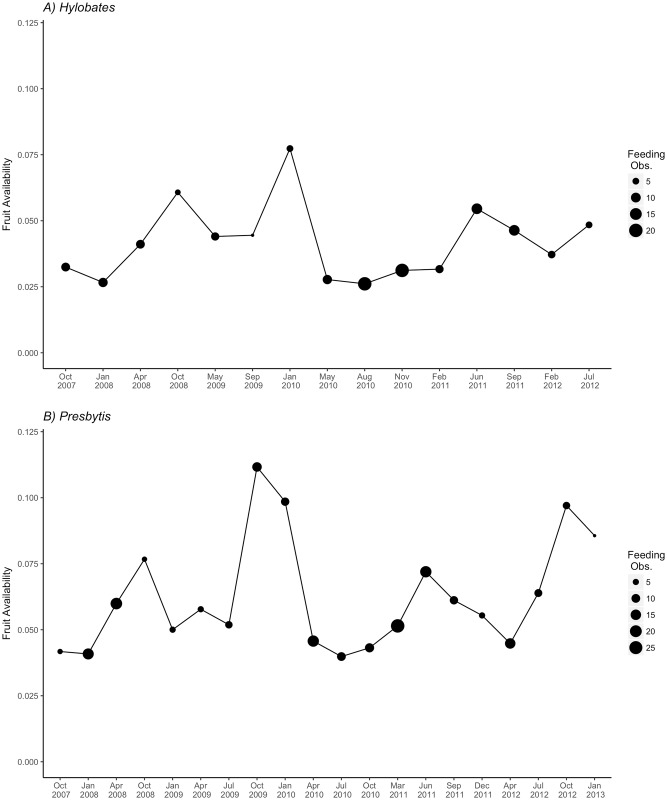
Plot of general forest productivity for gibbons (A: the proportion of stems in the forest bearing mature and ripe fruit) and leaf monkeys (B: proportion of stems bearing immature, mature and ripe fruit). There was substantial variation in fruit availability over the course of the study period. The size of the points represents the number of feeding observations for each three-month block.

The sampling period included a mast fruiting event from December 2009 though February 2010, during which plant reproductive output spiked. The proportion of plant stems fruiting in our plots during these three months ranged from 0.08 to 0.15 and averaged over 0.10, more than three times higher than lowest levels of productivity. This resulted in substantial variation in availability of food for both gibbons and leaf monkeys over the course of our study. Lumping of months to ensure adequate sample sizes for statistical analyses meant that variation in the proportion of fruiting stems in our 3-mo sampling blocks was more muted. For gibbons, the proportion of stems with mature and ripe fruit ranged from 0.026–0.077; for leaf monkeys the proportion of stems with immature, mature and ripe fruit ranged from 0.04–0.11 (fruit availability data provided in S3 Table). The sampling effort was not biased towards periods of high or low fruit availability (gibbons: N = 145, Mann Whitney U = 14, P = 0.1162; leaf monkeys: N = 219, Mann Whitney U = 32, P = 0.4319; Fig 1).

### Diet composition

**Fig 2:**
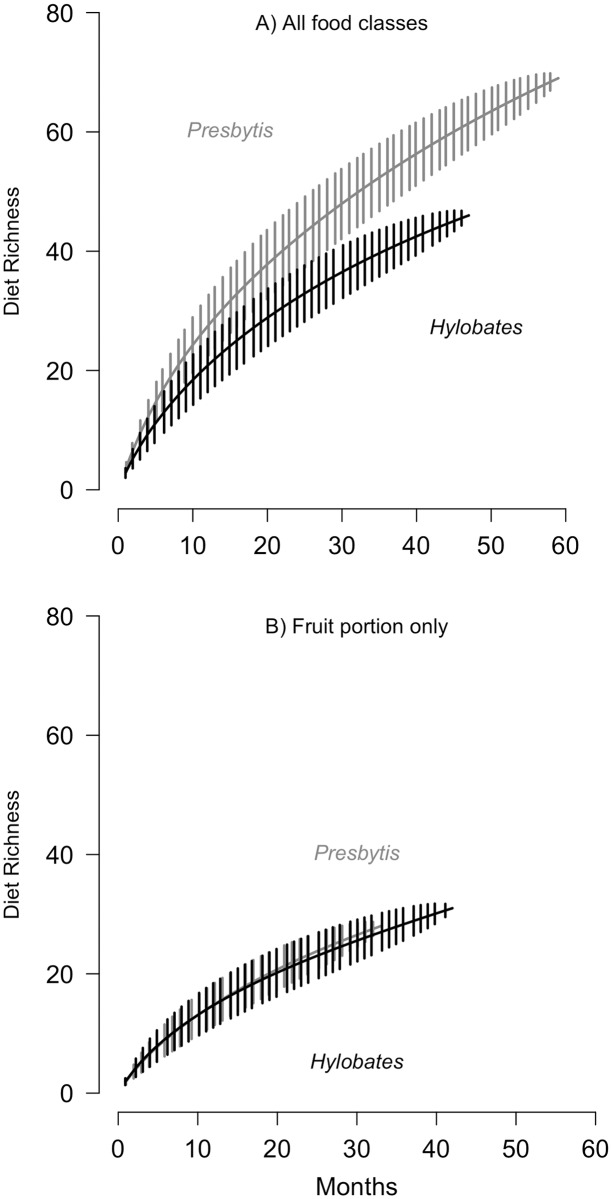
Rarefaction curves indicating the dietary richness of *P*. *rubicunda* and *H*. *albibarbis*. Dietary richness (number of genera) as a function of time (months of observation) for (A) all food classes and (B) only the frugivorous portion of the diet. Overall diet richness was greater for leaf monkeys (*P*. *rubicunda*) than for gibbons (*H*. *albibarbis*, A), but the species did not differ in the number of genera of fruits they consumed (B). Error bars represent +/- 1 standard deviation from the mean.

The species accumulation curves illustrate that leaf monkey diets had higher overall taxonomic richness than gibbon diets, when considering all food classes (Fig 2A). Neither curve reached an asymptote, reflecting that additional richness would be predicted with additional sampling. Nevertheless, the gibbon curve clearly separates from the leaf monkey curve and begins to flatten earlier, indicating that the differences in dietary diversity between the taxa are not the result of different sample sizes. In contrast to the overall diet, gibbons and leaf monkeys exhibited high similarity in the richness of the frugivorous portion of the diet (Fig 2B). Therefore, the higher overall dietary richness of leaf monkey diets is due to food classes other than fruit.

Leaf monkey diets were more diverse than gibbon diets both in terms of the total number of taxa fed upon and the distribution of feeding observations across food classes (Table 1). Leaf monkey diets were composed of 31.1% leaves (N = 68 observations), 25.6% fruits (N = 56), 26% seeds (N = 57), 8.2% figs (N = 18), 6.4% flowers (N = 14), and 2.7% (N = 6) unidentified items, whereas gibbon diets were composed of 50% fruits (N = 72), 20% figs (N = 29), 9.7% flowers (N = 14), 9.7% leaves (N = 14), and 6.9% (N = 10) unidentified items. Leaf monkeys consumed food sources from 69 genera in 37 families while gibbons consumed them from 46 genera in 40 families. Gibbons and leaf monkeys used different food classes from different genera. Leaf monkeys ate 28 genera of leaves, 13 genera of flowers, 32 genera of seeds, and 29 genera of fruits. Gibbons had a narrower dietary breadth, consuming 9 genera of leaves, 9 genera of flowers, 5 genera of seeds, and 31 genera of fruits.

**Table 1:** Contribution of various food classes and dietary overlap measures for gibbon and leaf monkey diets.

FOOD CLASSES							
	Leaf	Flower	Seed	Fruit (non-fig)	Fig	Unknown	Total
**Leaf Monkeys**							
Feeding Observations	68	14	57	56	18	6	219
Percentage of Diet	31.1%	6.4%	26.0%	25.6%	8.2%	2.7%	100%
Number of Genera	28	13	32	29	1	6	69
Dietary Overlap with Gibbons	0.09	0.29	0.04	0.46	1	NA	0.32
	Leaf	Flower	Seed	Fruit (non-fig)	Fig	Unknown	Total
**Gibbons**							
Feeding Observations	14	14	6	72	29	10	145
Percentage of Diet	9.7%	9.7%	4.1%	50.0%	20.0%	6.9%	100%
Number of Genera	9	9	5	31	1	7	46
Dietary Overlap With Leaf Monkeys	0.42	0.29	0.33	0.36	0.62	NA	0.48

### Dietary overlap

Our measure of dietary overlap included the number of genera consumed in common and the relative importance of each item in the diet, producing an index that permits asymmetry in dietary overlap measures. From the gibbon’s perspective, there was higher overall dietary overlap (when genera from all food classes were included) than from the leaf monkey’s perspective (0.48 vs. 0.32 overlap, respectively). This asymmetry is exemplified in the dietary overlap measures for the food class figs. From the leaf monkey perspective, the fig dietary overlap was 1; from the gibbon’s perspective it was 0.62. This asymmetry reflects differences in the importance of figs for the two primate species. In the fruit portion of the diet, leaf monkeys exhibited higher dietary overlap with gibbons (0.46) then vice versa (overlap from the gibbon’s perspective = 0.36), a pattern that was reversed in the leaf portion of the diet, with leaf monkeys exhibiting lower dietary overlap with gibbons (0.09) than gibbons with leaf monkeys (0.42; Table 1). Leaf monkeys also had lower dietary overlap with gibbons in terms of seeds consumed (0.04) than gibbons had with leaf monkeys (0.33).

### Dietary selectivity

Gibbons and leaf monkeys differed in the genera of fruit-bearing plants they consumed. For gibbons, 14 genera each contributed ≥ 1% of the total diet; 27% of all feeding observations came from one genus, *Ficus* (N = 29). Other important genera for gibbons were *Artabotrys* (Moraceae; 9.3%, N = 10), *Syzygium* (Myrtaceae; 8.4%, N = 9), *Willughbeia* (Apocynaceae; 5.6%, N = 6), and *Diospyros* (Ebenaceae; 4.7%, N = 5). Twenty-one genera were important food sources for leaf monkeys, each comprising ≥ 1% of the diet. The important foods sources for leaf monkeys were *Ficus* (Moraceae; 13.9%, N = 18), *Strombosia* (Olacaceae; 8.5%, N = 11), *Hydnocarpus* (Achariaceae; 7.8%, N = 10), *Xanthophyllum* (Polygalaceae; 7%, N = 9), *Artocarpus* (Moraceae; 5.5%, N = 7) and *Strychnos* (Loganiaceae; 5.5%, N = 7). Note that sample sizes are different for dietary composition and feeding selectivity, as we included raw counts of feeding observations for dietary composition, but included only observations of fruiting genera for the selectivity analyses.

**Fig 3:**
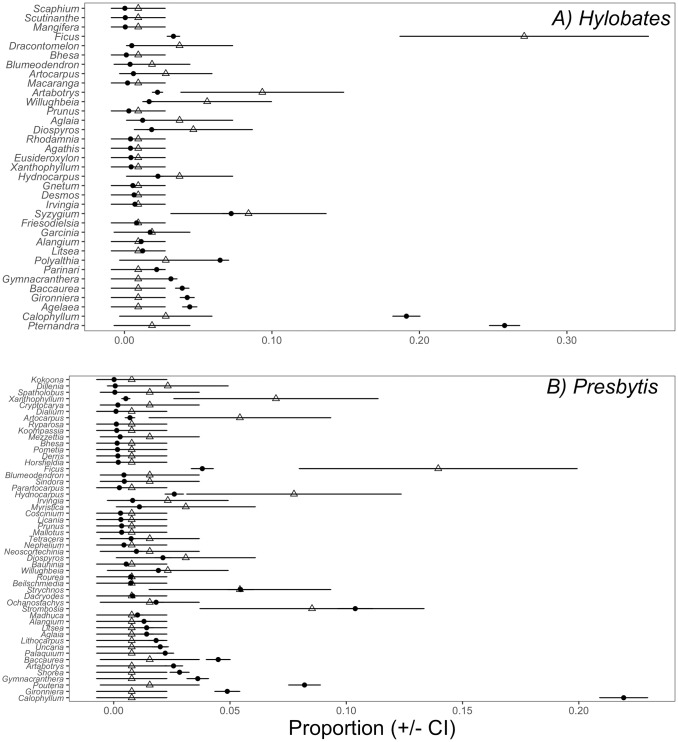
Feeding selectivity in gibbons (3a) and leaf monkeys (3b). Closed circles indicate availability (proportion of fruiting observations of genus *i*, out of total fruiting observations), open triangles indicate use (proportion of the diet), and error bars are 95% confidence intervals. Non-overlapping confidence intervals indicate positive selectivity (when triangles (use) are to the right of circles (availability)) or avoidance (when triangles (use) are to the left of the circles (availability)). Genera are listed from top to bottom in decreasing order of absolute selectivity values.

The differences between gibbon and leaf monkey diets were also evident in the preference for different genera (Gibbons Fig 3a; Leaf monkeys Fig 3b). Gibbons preferred two and avoided six out of the 35 genera on which they were observed to feed at least once, whereas leaf monkeys preferred four genera and avoided five of the 51 fruiting genera they consumed. Gibbons fed on *Ficus* (MSRs: 0.093) and *Artabotrys* (MSRs: 0.271) more than predicted based on their availability, and leaf monkeys preferred *Artocarpus* (MSRs: 0.054), *Ficus* (MSRs: 0.140), *Hydnocarpus* (MSR: 0.078) and *Xanthophyllum* (MSRs: 0.070). Gibbons avoided *Agelaea* (MSRs: 0.009), *Baccaurea* (MSR: 0.009), *Calophyllum* (MSRs: 0.028), *Gironniera* (MSRs: 0.009), *Gymnacranthera* (MSR: 0.009) and *Pternandra* (MSRs: 0.019). Leaf monkeys avoided *Baccaurea* (MSRs: 0.016), *Calophyllum* (MSR: 0.008), *Gironniera* (MSRs: 0.008), *Gymnacranthera* (MSR: 0.008) and *Pouteria* (MSRs: 0.016). We provide a complete list of standardized MSR values for both gibbons and leaf monkeys in S4 Table.

### Dietary composition as a function of fruit availability

Fruit availability was in the top model as a predictor of resource use for all classes of foods for both primates, with the exception of leaves (gibbons) and flowers (gibbons and leaf monkeys). The responses of gibbons and leaf monkeys to variation in fruit availability were qualitatively similar within food classes. We found that as overall fruit availability increased, the consumption of leaves and figs declined, while the consumption of fruit and seeds increased. For gibbons, the intercept only null model was the top model for leaf consumption (30% model weight), and this was also the case for flowers (28% model weight). For leaf monkeys, the strongest predictor of flower consumption was rainfall (27% model weight; for a complete list of models tested, model weights and delta AIC values see S5 Table).

**Fig 4:**
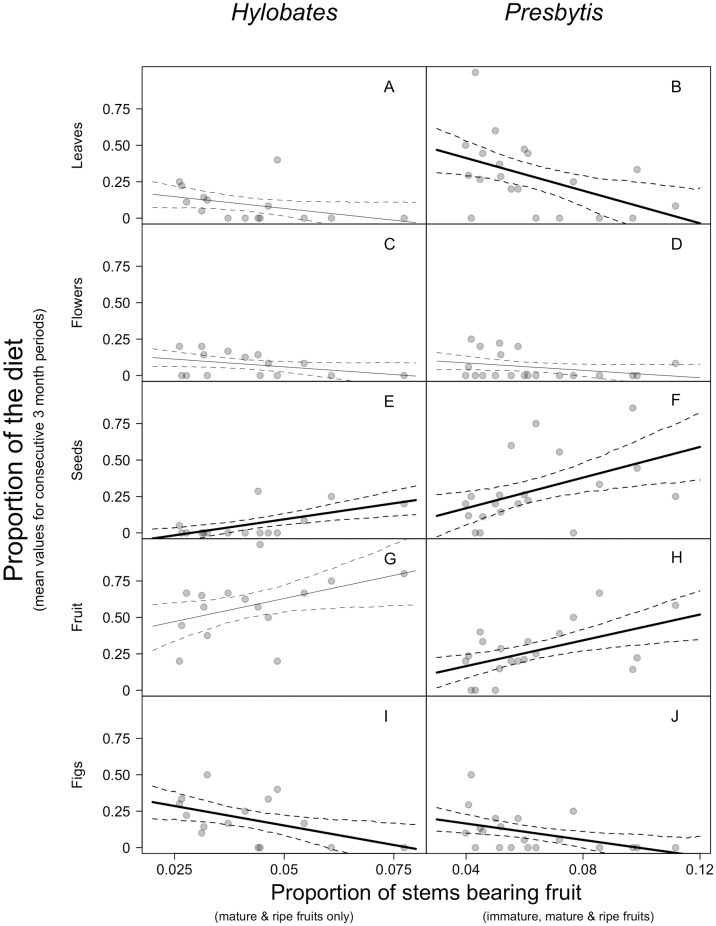
Proportions of food classes in the diet plotted against the proportion of stems in the forest bearing fruit. Data points show values for three-month blocks. Rows indicate dietary components comprising leaves (A, B), flowers (C, D), seeds (E, F), fruit (G, H), and figs (I, J). Note that estimates of fruit availability (x-axis in all plots) are based on different calculations for the two species: plots for *H*. *albibarbis* (left column) depict the proportion of stems bearing mature and ripe fruit only, whereas plots for *P*. *rubicunda* (right column) depict the proportion of stems bearing immature mature, and ripe fruits. Lines are ordinary least squares regression lines and standard errors (solid and dotted lines, respectively). Differences in sample size reflect differences in the number of three-month blocks with feeding observations from gibbons (n = 15) and leaf monkeys (n = 20). Bold regression lines indicate that fruit availability was a predictor in the top model for that plant part.

For the other food classes, fruit availability was the only predictor included in the top models. The slope estimate for the model of leaf consumption was negative (32% model weight; Fig 4B), meaning as the percentage of trees in the forest with fruit increased there was a decrease in the proportion of leaves in the diet. The proportion of figs in the diet also decreased in the diets of leaf monkeys (23% model weight; Fig 4J) and gibbons (31% model weight; Fig 4I) as fruit availability increased.

The pattern was reversed in the models of seed consumption, with fruit availability having a positive slope in models for both leaf monkeys (34% model weight; Fig 4F) and gibbons (44% model weight; Fig 4E). We also found a positive effect of fruit availability on fruit consumption for leaf monkeys (47% model weight; Fig 3H). But for gibbons, the best predictor of fruit consumption was availability of flowers (22% model weight). The proportion of fruit in the diet for gibbons was generally greater than 50% even during periods of low availability (Fig 4G), while this proportion rarely exceeded 50% in leaf monkey diets and only then during periods of high availability (Fig 4H).

## Discussion

We report the results of a long-term comparative study of the feeding ecology of two sympatric primates. In the masting Dipterocarp forests of Gunung Palung National Park in Indonesia, leaf monkeys had higher dietary richness and diversity than gibbons, which is likely due to leaf monkeys’ physiological ability to process and digest a broader range of foods. We analyzed resource selection from two perspectives: 1) by genus in the selectivity analyses, and 2) and by food class to investigate how these primates alter their diets in response to variation in fruit availability. The key distinction between these two approaches was that the food class analyses included changes in food availability over time, whereas the selectivity analyses did not; selectivity analyses included only measures of the commonness of stems. Despite the notable differences in dietary richness, both primates exhibited similar responses to variation in fruit availability. When fruit availability decreased, gibbons and leaf monkeys incorporated more leaves and figs into their diets, and when fruit availability increased they consumed more fruit and seeds. Below we briefly consider strengths and limitations of our methods, discuss how our results relate to what has been found for other populations of the same species in different habitats, and note how the results of our study fit more broadly in the context of other comparative studies of primate feeding ecology. Finally, we draw distinctions between selectivity analyses that are static (i.e., time invariant) and dynamic dietary composition analyses that incorporate temporal fluctuations in food availability. Using figs as an example, we discuss how a food source can paradoxically be identified as both a preferred and a fallback food, and highlight the importance of taking variation in food availability into account when characterizing primate diets.

### Methodological considerations and limitations

We acknowledge that our sample sizes for feeding data are smaller than for most studies of primate feeding ecology, even those that are conducted over much shorter periods than ours. Our sampling methods were a deliberate choice to ensure statistical independence, avoid pseudoreplication, and sample the population. Primate feeding data are typically collected during extended follows of focal animals and many feeding observations are recorded from the same group, often the same individual, on a single day. In addition, one or a small number of groups are typically sampled. These typical methods present challenges for appropriate statistical analysis, interpretation, and extrapolation beyond the sampled groups. Our methods place a primacy on ensuring statistical independence of feeding observations and avoiding biases associated with sampling few groups or habitat types. We note that while our sample sizes are small, they are unbiased, so the effects of small sample size are primarily on the width of confidence intervals and not the mean model results or general trends that we detected.

While we consider our data to be an accurate representation of the feeding behavior of the populations of the two species we studied, certain limitations warrant consideration. For example, most of our feeding observations were made before noon, and there is evidence that some primate species alter their food intake over the course of the day. Spider monkeys (*Ateles geoffroyi*) in Santa Rosa National Park, Costa Rica, eat more fruit in the mornings, and more leaves during midday and in the evening [55]. Both siamangs (*Symphalangus syndactylus*) and lar gibbons (*Hylobates lar*) in Peninsular Malaysia show a similar pattern where they consume more leaves later in the day [56]. If gibbons and leaf monkeys exhibit similar diurnal variation at our site, then our over-sampling of the morning would have biased our results. Sex and age can also have important influences on primate dietary composition. For example, in mountain gorillas (*Gorilla beringei)*, females and growing juveniles eat more food, and consume more protein per kilogram of body mass, than males [57]. Significant differences in dietary composition between age and sex classes have also been described for sifakas (*Propithecus verreauxi* [58]), green monkeys (*Cercopithecus sabaeus* [59]), and snub-nosed monkeys (*Rhinopithecus roxellana* [60]). We collected feeding observations from the first individual detected along the transect line or a randomly selected focal animal, regardless of age or sex class, so there are no inherent biases in our methods towards certain types of individuals. Nevertheless, it is possible that certain age or sex classes are more easily detected along transects and would therefore be oversampled in the data. We have not detected significant differences in feeding observations from transects (which are potentially subject to this bias) and randomly-selected focal follows (which are not [41]), but note that our small sample sizes mean that our power to reject the null model of no difference is limited.

### Dietary diversity, richness and overlap

We showed that gibbon and leaf monkey dietary overlap was asymmetrical and varied depending on the plant part consumed. Our results suggest that these primates are potentially important, albeit asymmetrical, food competitors, which is consistent with previous studies [40]. For example, dietary overlap for leaves and seeds was high from the gibbons’ perspective but low from the leaf monkeys’ perspective. This pattern was reversed for the dietary overlap for fruit, with leaf monkeys having a slightly higher measure of dietary overlap with gibbons than vice versa. Overall dietary overlap was higher from the gibbons’ perspective than from the leaf monkeys’ because leaf monkeys consumed a more diverse diet than gibbons did.

Our dietary overlap analyses did not account for variation in resource availability or differences in habitat types due to limitations in sample size, but both these factors could potentially influence dietary overlap measures. For example, in three sympatric primate species (*Ateles geoffroyi*, *Alouatta palliata*, and *Cebus capucinus*) in Santa Rosa National Park, Costa Rica, there was substantial variation in monthly dietary overlap measures [61]. The authors propose that the high dietary flexibility of these primate species, along with variation in dietary overlap, means that feeding competition may only be an intermittent selective force, occurring on a supra-annual basis. Data from our site demonstrate that the different forest types support different densities of gibbons and leaf monkeys [24], and that degree of resource overlap between gibbons and other vertebrate frugivores varies substantially between peat swamps and other forest types [40]. Importantly, our results of dietary overlap were consistent with results from other aspects of this study, showing that for the frugivorous portion of the diet, gibbons and leaf monkeys have similar measures of dietary overlap and dietary richness, but differences in dietary overlap and richness occur in the leaf and seed portion of the diet.

Behavioral mechanisms, such as the incorporation of a broad range of foods in the diet, may allow folivorous primates such as leaf monkeys to reduce the amount of toxins consumed (the “diet-breadth trade-off” hypothesis [62]). By incorporating more varied food sources into their diets than gibbons, leaf monkeys at our site may mitigate the potential buildup of specific toxins from seeds and leaves, leading to greater diversity in the non-frugivorous portion of their diets. Fruits are easy to process and digest [63] while leaves often contain antifeedants and other compounds that can be toxic to primates in large amounts [31]. Unripe fruits are heavily chemically defended, possessing relatively high levels of antifeedants [64,65] and seeds also contain secondary compounds, such as strychnine and alkaloids, that can be toxic in large quantities [66]. Our results are consistent with a recent study investigating dietary flexibility of two lemur genera, the omnivorous *Eulemur*, with the more folivorous *Propithecus*, where anatomical adaptations to digest fiber allowed for increased dietary breadth in *Propithecus* [67].

### Dietary selectivity

The results of the selectivity analyses provide further evidence that gibbons have a narrower diet than leaf monkeys, at least in the masting forests of CPRS. In line with our predictions, leaf monkeys preferred more genera and avoided fewer genera than gibbons. Both gibbons and leaf monkeys selected figs (genus *Ficus*) more than predicted based on their availability, when temporal variation was not considered. Interestingly, gibbons and leaf monkeys both avoided four of the same genera: *Baccaurea*, *Calophyllum*, *Gironniera* and *Gymnacranthera*. These genera exhibit relatively low synchrony similar to figs, meaning that they are consistently available in the environment. For gibbons, the most important predictors of resource use are overall abundance of the genus, as well as the consistency of fruit availability [41]. Although these four genera were relatively abundant, and consistently available, gibbons and leaf monkeys both avoided them, most likely because they are of low quality for primates (e.g., *Calophyllum* is a highly tannic, bat-dispersed fruit; *Gymnacranthera* produces thin, lipid-rich but sugar-poor pulp that targets dispersal by hornbills). It is important to note that use or importance of a food item in a primate diet is different than preference [9], and genera that are classified as avoided may still constitute a substantial portion of the primate’s diet. This is because very common items may be consumed fairly often (making them important), but eaten much less often than would be predicted based on their availability (making them avoided).

Despite methodological differences, both Marshall et al. [40] and our selectivity analyses returned qualitatively similar results for gibbons. For example, both studies ranked the liana *Artabotrys* as being the most highly selected genus for gibbons. *Artabotrys* exhibits low synchrony, so it is consistently available [41]. The high selectivity score for *Artabotrys* in both our study and previous studies indicates that gibbons are consuming this genus more than predicted based on availability, which is probably because it produces relatively high quality, sugar-rich fruits that are easy to process, as well as the fact that it is often available during periods of otherwise low food availability [41,68]. Our selectivity results are also similar to those of gibbon hybrids (*Hylobates muelleri* x *albibarbis*) in the Barito Ulu research area, Central Kalimantan, Indonesia [69].

### Response to variation in resource availability

Both gibbons and leaf monkeys experienced dramatic fluctuations in resource availability during the study period. Fruit availability varied by more than an order of magnitude among months, from less than 1% of stems to over 15% of stems bearing fruit—to place these numbers in context, note that majority of trees that we have monitored in our plots never fruited in a seven-year period that included two mast fruiting events [18]. Extreme fluctuations in fruit availability, which are unpredictable from the primate’s perspective, are important forces shaping primate behavior, ecology and morphology [8,9,25,28]. It is therefore crucial to capture the full range of variation in fruit availability when studying primate diets. Our study encompassed at least one masting event- two for leaf monkeys due to random variation in sampling of the different primate species- which allowed us to compare the primate diets over the full range of resource availability they are likely to experience.

Despite the fact that gibbons and leaf monkeys have distinctly different diets, their dietary response to decreased fruit availability was qualitatively similar. Both primates showed increased consumption of leaves when fruit was less abundant. A similar pattern was seen for figs, with both primates increasing their fig consumption when fruit was less abundant, and eating very few figs during periods of high fruit availability. The patterns of leaf and fig consumption exhibited by gibbon and leaf monkeys indicate that they utilized these foods as fallback foods, as reported in analyses of earlier data sets at CPRS [14,24,25]. We discuss how figs can be paradoxically identified as preferred (over-selected) and as fallback foods below.

Our results are consistent with past studies conducted on leaf monkeys in masting forests on Borneo. In areas where resource availability is highly variable, leaf monkeys eat more leaves during periods of low fruit availability, but incorporate fruit into the diet when it is available [70]. This dietary switching has not been shown to occur in non-masting forests. For example, leaf monkeys were shown to have a higher degree of frugivory in non-masting peat swamp forests of Sabangau, Central Kalimantan, Indonesia than those reported by other researchers in masting dipterocarp forests of Gunung Palung [39], Sepilok [71], Tanjung Puting [72] and Danum Valley [70], and the amount of fruit included in the diet was not influenced by changes in overall fruit availability [73]. Leaf monkeys did not incorporate low quality foods into their diets in the non-masting peat forests, as fruit was consistently available year-round. Although peat swamp forests are present at our study site, we excluded peat swamp forest from our investigation of the influence of fruit availability on dietary composition because combining feeding observations from masting and non-masting forests that were phenologically unsynchronized would have confounded the analysis. Our results supporting the occurrence of dietary shifts in response to food availability suggest that differences in dietary strategies used in peat swamp forests versus dipterocarp forests may be substantial.

Both primate species in our study showed a marked increase in fruit consumption when fruit availability increased, and even during periods of fruit scarcity, fruit comprised at least 50% of gibbon diets, which is consistent with studies at other sites [68,74] and previous studies at CPRS [14,24,40]. The same pattern was seen with seed consumption, and this is most likely because seed and fruit availability are intrinsically linked. The top model for gibbon leaf consumption did not include total fruit availability. This may be because gibbons do not consume many leaves, regardless of fruit availability, as leaves rarely comprised more than 25% of their feeding observations. It is also possible that with more sampling, a stronger relationship between fruit availability and leaves would emerge.

Our results are consistent with other comparative primate studies. For example, three sympatric new world primates (*Callimico goeldii*, *Saguinus fuscicollis and S*. *labiatus*) showed high degree of dietary overlap during periods of high fruit availability when a few abundant fruit species dominated their diets, but during periods of low fruit availability their diets diverged, and they incorporated more fungus (*C*. *goeldii)*, nectar (*S*. *labiatus*) or arthropods (*S*. *fuscicollis*) into their diets [75]. Exploitation of different food resources during periods of resource scarcity, as well differences in foraging heights, may allow these closely related primates to live in sympatry. Tutin and Fenandez [76] compared the diets of sympatric chimpanzees and gorillas at Lope, Gabon and found a high degree of dietary overlap, particularly for fruits. Importantly, they found that the diets of these two primates diverged most during periods of fruit scarcity, and that gorillas incorporated more leaves, stems and bark into their diets, while chimpanzees maintained high levels of frugivory even during times when fruit was scarce. Dietary divergence during periods of resource scarcity potentially allows primates to mitigate the effects of feeding competition, and the authors interpreted dietary divergence as evidence of niche separation in these two sympatric primates. In our study, we provide further evidence that dietary divergence is an important mechanism in which closely-related, sympatric primate species may mitigate feeding competition.

### The importance of figs

Figs have been classified as an important food for primates throughout the tropics, and are often fallback foods, due to their relatively constant availability and increased representation in the diet when other fruit is scarce [19,28], but in some sites figs have been identified as “preferred” or over-selected [77,78]. Differences between sites have been attributed to differences in the quality of non-fig fruits, with figs being fallback foods in sites where there are other high-quality fruits, and figs being preferred foods in sites where high-quality non-fig fruits are lacking [78]. In our study, both gibbons and leaf monkeys showed a marked decrease in fig consumption when fruit availability increased. In addition, the proportion of figs included in gibbon and leaf monkey diets was similar (approximately 0.25 during periods of low fruit availability but almost zero during periods of high fruit availability).

We analyzed figs from two perspectives: 1) as a genus (*Ficus*) in the selectivity analyses which did not take into account temporal variation of food availability, and 2) as a food class when investigating the proportion of stems included in the diet, which did take into account changes in food availability over time. In the selectivity analyses, the genus *Ficus* was an important and preferred food because gibbons select figs more than would be expected based on the stem density of figs. Use of the food class “figs” decreased with an increase in overall fruit availability. In this context, figs were a fallback food because they were eaten in periods of low fruit availability, and consumption was inversely correlated with preferred food (fruit) availability. Thus, as a food class, figs conformed to the definition of a fallback food, but in the selectivity analyses that excluded temporal variation in food availability, gibbons preferred figs. This difference occurred because figs were more consistently available than most genera and gibbons disproportionately feed on genera that are consistently available over time [41].

Previous research from our study site found that the number of gibbon feeding observations on figs was negatively correlated with availability of ripe fruit, and thus figs were a fallback food for gibbons [14]. We present the results of over five additional years of new feeding observations and find a consistent result that the proportion of figs in gibbon diets decrease as fruit availability increased. Our study therefore substantiates the earlier finding that figs are an important food class for gibbons during periods of low fruit availability, and highlights that this pattern is stable over long periods.

### Implications for conservation

Characterizing primate diets has important conservation implications. For example, preferred and fallback foods may be important factors influencing primate population density [14]. Gibbon population density at Gunung Palung National Park is highly correlated with the abundance of their main fallback food, while leaf monkey population density is highly correlated with the abundance of their preferred foods [25]. This trend may not be consistent across regions, as colobine biomass was correlated with proportion of trees that are legumes (Fabaceae), which are generally preferred foods, in Asia but not in Africa [29]–although differences in sampling intensity, study duration and study design may also contribute to these differences. This potential for regional variation in preferred and fallback foods, as well as the implications for primate ecology, highlights the needs for intensive sampling of a broad range of diets of various primate species and populations.

In addition, anthropogenic climate change is and will continue to impact plant reproductive phenology [79]. In the face of a changing climate, especially in hyper-variable dipterocarp forests where masting is correlated with El Niño events [17], long-term data sets can provide reference points for primate feeding ecology, which may be useful to assess how climate change affects primate species and their food resources. Feeding ecology studies can provide insights into the range of resources used by primates, and may allow for predictions about how habitat disturbance, such as selective logging of primate food trees, will influence their ecology and likelihood of persistence. Feeding ecology studies can also be used to inform conservation actions for specific populations and metapopulations [80]. For example, an in-depth knowledge of primate feeding ecology can be used in habitat restoration projects, when the goal is to minimize periods of resource scarcity for primates in restored habitats [81].

### Conclusions

This study contributes to existing knowledge of gibbon and leaf monkey natural history, diets and responses to variation in resource availability. These two sympatric primate species, despite having quantitatively different diets, exhibit similar feeding strategies when faced with variation in food availability. We show that during times of high fruit availability, both primates incorporate a high proportion of fruit in their diet, and during periods of low fruit availability they incorporate more figs and leaves. In addition, our results show that despite differences in diet and life history, both primates utilized figs as a fallback food, providing further support for the importance of figs as a fallback food for a broad range of primates. Additional comparative studies on sympatric primates in different regions will improve understanding of how primates alter their diets in response to resource scarcity and show if our results apply more broadly.

## Supporting information

S1 Table List of gibbon feeding observations by genus and plant part consumed. (CSV)

S2 Table List of leaf monkey feeding observations by genus and plant part consumed. (CSV)

S3 Table Summary of fruit availability, temperature and rainfall during three month blocks for gibbons and leaf monkeys over the course of the study period. Raw counts of stems are based on reproductive status using the following definitions: B: containing flower buds (i.e., developing flowers visible, but no flowers at anthesis); F: mature flowers (i.e., at least one flower on the tree as at anthesis); I: immature fruit (i.e., fruits with undeveloped seeds); M: mature fruits (i.e., unripe fruits that are full-sized and have fully developed and hardened seeds); R: mature fruit (i.e., ripe fruit; fruits fully mature, usually signaled by a change in color or softness).; X: reproductively inactive.
(CSV)

S4 Table Selectivity table for gibbons and leaf monkeys. Larger selectivity values indicate preferred genera, whereas values closer to zero indicate less preferred genera. Use values are the total number of feeding observations on genus *i*. Availability values were calculated as the total number of stems of genus *i* that were observed to fruit during the study period. Bold and starred selectivity values indicate non-overlapping confidence intervals (see text for explanation) and therefore statistical significance (P < 0.05).
(DOCX)

S5 Table Complete list of models used in AICc model comparison. Each model represents a specific prediction (e.g. model 1: gibbons will feed more on figs when there is low fruit availability).
(DOCX)
