# Supplementary material for: Dietary diversity, feeding selectivity, and responses to fruit scarcity of two sympatric Bornean primates (Hylobates albibarbis and Presbytis rubicunda rubida)
Source: PLoS One. 2017 Mar 9;12(3):e0173369. doi: 10.1371/journal.pone.0173369 (PMC5344392; doi:10.1371/journal.pone.0173369)
Supplement: S4 Table — Larger selectivity values indicate preferred genera, whereas values closer to zero indicate less preferred genera. Use values are the total number of feeding observations on genus i. Availability values were calculated as the total number of stems of genus i that were observed to fruit during the study period. Bold and starred selectivity values indicate non-overlapping confidence intervals (see text for explanation) and therefore statistical significance (P < 0.05). (DOCX) [file pone.0173369.s004.docx]

**Table S1. Selectivity table for gibbons and leaf monkeys.** Larger selectivity values indicate preferred genera, whereas values closer to zero indicate less preferred genera. Use values are the proportion of feeding observations of genus *i* over the total number of feeding observations, and were calculated for genera on which there was at least one feeding observation. Availability values included stems that were observed to fruit at least once. See text for equations used to calculate values. Bold and starred selectivity values indicate non-overlapping error bars and statistical significance (P < 0.05).

|  | ***Gibbons*** |  |  | ***Leaf Monkeys*** |  |  |
| --- | --- | --- | --- | --- | --- | --- |
| **Genus** | **Selectivity** | **Use** | **Availability** | **Selectivity** | **Use** | **Availability** |
| *Agathis* | 0.009 | 0.009 | 0.002 | ~ | ~ | ~ |
| *Agelaea* | **0.009*** | 0.009 | 0.057 | ~ | ~ | ~ |
| *Aglaia* | 0.037 | 0.037 | 0.013 | 0.008 | 0.008 | 0.013 |
| *Alangium* | 0.009 | 0.009 | 0.012 | 0.008 | 0.008 | 0.014 |
| *Artabotrys* | **0.093*** | 0.093 | 0.025 | 0.008 | 0.008 | 0.021 |
| *Artocarpus* | 0.028 | 0.028 | 0.002 | **0.054*** | 0.054 | 0.007 |
| *Baccaurea* | **0.009*** | 0.009 | 0.045 | **0.016*** | 0.016 | 0.054 |
| *Bauhinia* | ~ | ~ | ~ | 0.008 | 0.008 | 0.004 |
| *Beilschmiedia* | ~ | ~ | ~ | 0.008 | 0.008 | 0.007 |
| *Bhesa* | 0.009 | 0.009 | 0.001 | 0.008 | 0.008 | 0.001 |
| *Blumeodendron* | 0.019 | 0.019 | 0.004 | 0.016 | 0.016 | 0.004 |
| *Calophyllum* | **0.028*** | 0.028 | 0.200 | **0.008*** | 0.008 | 0.218 |
| *Coscinium* | ~ | ~ | ~ | 0.008 | 0.008 | 0.002 |
| *Cryptocarya* | ~ | ~ | ~ | 0.016 | 0.016 | 0.002 |
| *Dacryodes* | ~ | ~ | ~ | 0.008 | 0.008 | 0.007 |
| *Derris* | ~ | ~ | ~ | 0.008 | 0.008 | 0.001 |
| *Desmos* | 0.009 | 0.009 | 0.006 | ~ | ~ | ~ |
| *Dialium* | ~ | ~ | ~ | 0.008 | 0.008 | 0.001 |
| *Dillenia* | ~ | ~ | ~ | 0.023 | 0.023 | 0.001 |
| *Diospyros* | 0.047 | 0.047 | 0.021 | 0.031 | 0.031 | 0.020 |
| *Dracontomelon* | 0.037 | 0.037 | 0.006 | ~ | ~ | ~ |
| *Eusideroxylon* | 0.009 | 0.009 | 0.005 | ~ | ~ | ~ |
| *Ficus* | **0.271*** | 0.271 | 0.032 | **0.140*** | 0.140 | 0.031 |
| *Friesodielsia* | 0.009 | 0.009 | 0.009 | ~ | ~ | ~ |
| *Garcinia* | 0.019 | 0.019 | 0.018 | ~ | ~ | ~ |
| *Gironniera* | **0.009*** | 0.009 | 0.055 | **0.008*** | 0.008 | 0.066 |
| *Gnetum* | 0.009 | 0.009 | 0.006 | ~ | ~ | ~ |
| *Gymnacranthera* | **0.009*** | 0.009 | 0.035 | **0.008*** | 0.008 | 0.040 |
| *Horsfieldia* | ~ | ~ | ~ | 0.008 | 0.008 | 0.002 |
| *Hydnocarpus* | 0.037 | 0.037 | 0.028 | **0.078*** | 0.078 | 0.022 |
| *Irvingia* | 0.009 | 0.009 | 0.008 | 0.023 | 0.023 | 0.007 |
| *Kokoona* | ~ | ~ | ~ | 0.008 | 0.008 | 0.000 |
| *Koompassia* | ~ | ~ | ~ | 0.008 | 0.008 | 0.001 |
| *Licania* | ~ | ~ | ~ | 0.008 | 0.008 | 0.003 |
| *Lithocarpus* | ~ | ~ | ~ | 0.008 | 0.008 | 0.017 |
| *Litsea* | 0.009 | 0.009 | 0.009 | 0.008 | 0.008 | 0.012 |
| *Macaranga* | 0.009 | 0.009 | 0.002 | ~ | ~ | ~ |
| *Madhuca* | ~ | ~ | ~ | 0.008 | 0.008 | 0.011 |
| *Mallotus* | ~ | ~ | ~ | 0.008 | 0.008 | 0.003 |
| *Mangifera* | 0.009 | 0.009 | 0.001 | ~ | ~ | ~ |
| *Mezzettia* | ~ | ~ | ~ | 0.016 | 0.016 | 0.003 |
| *Myristica* | ~ | ~ | ~ | 0.031 | 0.031 | 0.012 |
| *Neoscortechinia* | ~ | ~ | ~ | 0.016 | 0.016 | 0.014 |
| *Nephelium* | ~ | ~ | ~ | 0.008 | 0.008 | 0.005 |
| *Ochanostachys* | ~ | ~ | ~ | 0.016 | 0.016 | 0.016 |
| *Palaquium* | ~ | ~ | ~ | 0.008 | 0.008 | 0.021 |
| *Parartocarpus* | ~ | ~ | ~ | 0.008 | 0.008 | 0.002 |
| *Parinari* | 0.009 | 0.009 | 0.017 | ~ | ~ | ~ |
| *Polyalthia* | 0.028 | 0.028 | 0.064 | ~ | ~ | ~ |
| *Pometia* | ~ | ~ | ~ | 0.008 | 0.008 | 0.001 |
| *Pouteria* | ~ | ~ | ~ | **0.016*** | 0.016 | 0.079 |
| *Prunus* | 0.009 | 0.009 | 0.003 | 0.008 | 0.008 | 0.004 |
| *Pternandra* | **0.019*** | 0.019 | 0.224 | ~ | ~ | ~ |
| *Rhodamnia* | 0.009 | 0.009 | 0.004 | ~ | ~ | ~ |
| *Rourea* | ~ | ~ | ~ | 0.008 | 0.008 | 0.006 |
| *Ryparosa* | ~ | ~ | ~ | 0.008 | 0.008 | 0.001 |
| *Scaphium* | 0.009 | 0.009 | 0.000 | ~ | ~ | ~ |
| *Scutinanthe* | 0.009 | 0.009 | 0.001 | ~ | ~ | ~ |
| *Shorea* | ~ | ~ | ~ | 0.008 | 0.008 | 0.026 |
| *Sindora* | ~ | ~ | ~ | 0.016 | 0.016 | 0.004 |
| *Spatholobus* | ~ | ~ | ~ | 0.016 | 0.016 | 0.000 |
| *Strombosia* | ~ | ~ | ~ | 0.085 | 0.085 | 0.130 |
| *Strychnos* | ~ | ~ | ~ | 0.054 | 0.054 | 0.044 |
| *Syzygium* | 0.084 | 0.084 | 0.065 | ~ | ~ | ~ |
| *Tetracera* | ~ | ~ | ~ | 0.016 | 0.016 | 0.006 |
| *Uncaria* | ~ | ~ | ~ | 0.008 | 0.008 | 0.016 |
| *Willughbeia* | 0.056 | 0.056 | 0.019 | 0.023 | 0.023 | 0.016 |
| *Xanthophyllum* | 0.009 | 0.009 | 0.004 | **0.070*** | 0.070 | 0.005 |
