# Supplementary material for: Dietary diversity, feeding selectivity, and responses to fruit scarcity of two sympatric Bornean primates (Hylobates albibarbis and Presbytis rubicunda rubida)
Source: PLoS One. 2017 Mar 9;12(3):e0173369. doi: 10.1371/journal.pone.0173369 (PMC5344392; doi:10.1371/journal.pone.0173369)
Supplement: S5 Table — Each model represents a specific prediction (e.g. model 1: gibbons will feed more on figs when there is low fruit availability). (DOCX) [file pone.0173369.s005.docx]

**Gibbons and Fig Use**

| **Model** | ***Structure*** |
| --- | --- |

| Zero | *Fig use ~ Intercept* |
| --- | --- |
| One | *Fig use ~ Fruit Availability* |
| Two | *Fig use ~ Seed Availability* |
| Three | *Fig use ~ Flower Availability* |
| Four | *Fig use ~ Fig Availability* |
| Five | *Fig use ~ Maximum mean temperature* |
| Six | *Fig use ~ Minimum mean temperature* |
| Seven | *Fig use ~ Rainfall* |
| Eight | *Fig use ~ Fruit Availability + Maximum mean temperature* |
| Nine | *Fig use ~ Fruit Availability + Maximum mean temperature+ Minimum mean temperature + Rainfall* |
| Ten | *Fig use ~ Seed Availability + Maximum mean temperature+ Minimum mean temperature + Rainfall* |
| Eleven | *Fig use ~ Flower Availability + Maximum mean temperature+ Minimum mean temperature + Rainfall* |
| Twelve | *Fig use ~ Fig Availability + Maximum mean temperature+ Minimum mean temperature + Rainfall* |

**Results of Model Selection for Fig Use**

| **Model** | **deltaAIC df Model weight** |
| --- | --- |

| One | 0 | 3 | 0.311 |
| --- | --- | --- | --- |
| Zero | 0.8 | 2 | 0.211 |
| Three | 1.7 | 3 | 0.136 |
| Two | 2.4 | 3 | 0.093 |
| Seven | 3.3 | 3 | 0.059 |
| Eight | 3.5 | 4 | 0.053 |
| Five | 3.8 | 3 | 0.047 |
| Six | 3.9 | 3 | 0.044 |
| Four | 3.9 | 3 | 0.043 |
| Nine | 11.7 | 6 | <0.001 |
| Eleven | 15 | 6 | <0.001 |
| Ten | 15.3 | 6 | <0.001 |
| Twelve | 17.4 | 6 | <0.001 |

**Gibbons and Leaf Use**

| **Model** | ***Structure*** |
| --- | --- |

| Zero | *Leaf use ~ Intercept* |
| --- | --- |
| One | *Leaf use ~ Fruit Availability* |
| Two | *Leaf use ~ Seed Availability* |
| Three | *Leaf use ~ Flower Availability* |
| Four | *Leaf Use ~ Fig Availability* |
| Five | *Leaf Use ~ Maximum mean temperature* |
| Six | *Leaf Use ~ Minimum mean temperature* |
| Seven | *Leaf Use ~ Rainfall* |
| Eight | *Leaf Use ~ Fruit Availability + Maximum mean temperature* |
| Nine | *Leaf Use ~ Fruit Availability + Maximum mean temperature+ Minimum mean temperature + Rainfall* |
| Ten | *Leaf Use ~ Seed Availability + Maximum mean temperature+ Minimum mean temperature + Rainfall* |
| Eleven | *Leaf Use ~ Flower Availability + Maximum mean temperature+ Minimum mean temperature + Rainfall* |
| Twelve | *Leaf Use ~ Fig Availability + Maximum mean temperature+ Minimum mean temperature + Rainfall* |

**Results of Model Selection for Leaf Use**

| **Model** | **deltaAIC df Model weight** |
| --- | --- |

| Zero | 0 | 2 | 0.296 |
| --- | --- | --- | --- |
| One | 0.8 | 3 | 0.2009 |
| Six | 1.6 | 3 | 0.1323 |
| Four | 2.9 | 3 | 0.0698 |
| Seven | 3 | 3 | 0.0672 |
| Three | 3 | 3 | 0.0647 |
| Two | 3.1 | 3 | 0.0615 |
| Five | 3.2 | 3 | 0.0603 |
| Eight | 3.9 | 4 | 0.043 |
| Nine | 8.7 | 6 | 0.0038 |
| Twelve | 15 | 6 | <0.001 |
| Ten | 15.1 | 6 | <0.001 |
| Eleven | 15.2 | 6 | <0.001 |

**Gibbons and Seed Use**

| **Model** | ***Structure*** |
| --- | --- |

| Zero | *Seed use ~ Intercept* |
| --- | --- |
| One | *Seed use ~ Fruit Availability* |
| Two | *Seed use ~ Seed Availability* |
| Three | *Seed use ~ Flower Availability* |
| Four | *Seed use ~ Fig Availability* |
| Five | *Seed use ~ Maximum mean temperature* |
| Six | *Seed use ~ Minimum mean temperature* |
| Seven | *Seed use ~ Rainfall* |
| Eight | *Seed use ~ Fruit Availability + Maximum mean temperature* |
| Nine | *Seed use ~ Fruit Availability + Maximum mean temperature+ Minimum mean temperature + Rainfall* |
| Ten | *Seed use ~ Seed Availability + Maximum mean temperature+ Minimum mean temperature + Rainfall* |
| Eleven | *Seed use ~ Flower Availability + Maximum mean temperature+ Minimum mean temperature + Rainfall* |
| Twelve | *Seed use ~ Fig Availability + Maximum mean temperature+ Minimum mean temperature + Rainfall* |

**Results of Model Selection for Seed Use**

| **Model** | **deltaAIC df Model weight** |
| --- | --- |

| Zero | 0 | 2 | 0.296 |
| --- | --- | --- | --- |
| One | 0.8 | 3 | 0.2009 |
| Six | 1.6 | 3 | 0.1323 |
| Four | 2.9 | 3 | 0.0698 |
| Seven | 3 | 3 | 0.0672 |
| Three | 3 | 3 | 0.0647 |
| Two | 3.1 | 3 | 0.0615 |
| Five | 3.2 | 3 | 0.0603 |
| Eight | 3.9 | 4 | 0.043 |
| Nine | 8.7 | 6 | 0.0038 |
| Twelve | 15 | 6 | <0.001 |
| Ten | 15.1 | 6 | <0.001 |
| Eleven | 15.2 | 6 | <0.001 |

**Gibbons and Flower use**

| **Model** | ***Structure*** |
| --- | --- |

| Zero | *Flower use ~ Intercept* |
| --- | --- |
| One | *Flower use ~ Fruit Availability* |
| Two | *Flower use ~ Seed Availability* |
| Three | *Flower use ~ Flower Availability* |
| Four | *Flower use ~ Fig Availability* |
| Five | *Flower use ~ Maximum mean temperature* |
| Six | *Flower use ~ Minimum mean temperature* |
| Seven | *Flower use ~ Rainfall* |
| Eight | *Flower use ~ Fruit Availability + Maximum mean temperature* |
| Nine | *Flower use ~ Fruit Availability + Maximum mean temperature+ Minimum mean temperature + Rainfall* |
| Ten | *Flower use ~ Seed Availability + Maximum mean temperature+ Minimum mean temperature + Rainfall* |
| Eleven | *Flower use ~ Flower Availability + Maximum mean temperature+ Minimum mean temperature + Rainfall* |
| Twelve | *Flower use ~ Fig Availability + Maximum mean temperature+ Minimum mean temperature + Rainfall* |

**Results of Model Selection for Flower use**

| **Model** | **deltaAIC df Model weight** |
| --- | --- |

| Zero | 0 | 2 | 0.2808 |
| --- | --- | --- | --- |
| One | 0.9 | 3 | 0.1773 |
| Five | 2 | 3 | 0.1046 |
| Seven | 2.3 | 3 | 0.0911 |
| Three | 2.7 | 3 | 0.0729 |
| Six | 2.7 | 3 | 0.0713 |
| Two | 2.9 | 3 | 0.0661 |
| Four | 3.2 | 3 | 0.0575 |
| Eight | 4.5 | 4 | 0.03 |
| Twelve | 4.7 | 6 | 0.0267 |
| Nine | 6.9 | 6 | 0.0089 |
| Eleven | 7.5 | 6 | 0.0065 |
| Ten | 7.6 | 6 | 0.0062 |

**Gibbons and Fruit use**

| **Model** | ***Structure*** |
| --- | --- |

| Zero | *Fruit use ~ Intercept* |
| --- | --- |
| One | *Fruit use ~ Fruit Availability* |
| Two | *Fruit use ~ Seed Availability* |
| Three | *Fruit use ~ Flower Availability* |
| Four | *Fruit use ~ Fig Availability* |
| Five | *Fruit use ~ Maximum mean temperature* |
| Six | *Fruit use ~ Minimum mean temperature* |
| Seven | *Fruit use ~ Rainfall* |
| Eight | *Fruit use ~ Fruit Availability + Maximum mean temperature* |
| Nine | *Fruit use ~ Fruit Availability + Maximum mean temperature+ Minimum mean temperature + Rainfall* |
| Ten | *Fruit use ~ Seed Availability + Maximum mean temperature+ Minimum mean temperature + Rainfall* |
| Eleven | *Fruit use ~ Flower Availability + Maximum mean temperature+ Minimum mean temperature + Rainfall* |
| Twelve | *Fruit use ~ Fig Availability + Maximum mean temperature+ Minimum mean temperature + Rainfall* |

**Results of Model Selection for Fruit use**

| **Model** | **deltaAIC df Model weight** |
| --- | --- |

| Three | 0 | 3 | 0.215 |
| --- | --- | --- | --- |
| Zero | 0.2 | 2 | 0.191 |
| One | 0.5 | 3 | 0.168 |
| Two | 0.7 | 3 | 0.154 |
| Four | 1.5 | 3 | 0.103 |
| Six | 2.7 | 3 | 0.057 |
| Five | 3.4 | 3 | 0.039 |
| Seven | 3.4 | 3 | 0.039 |
| Eight | 3.7 | 4 | 0.034 |
| Nine | 11.9 | 6 | <0.001 |
| Twelve | 13.8 | 6 | <0.001 |
| Eleven | 13.9 | 6 | <0.001 |
| Ten | 14.8 | 6 | <0.001 |

**Leaf Monkeys and Fig Use**

| **Model** | ***Structure*** |
| --- | --- |

| Zero | *Fig use ~ Intercept* |
| --- | --- |
| One | *Fig use ~ Fruit Availability* |
| Two | *Fig use ~ Seed Availability* |
| Three | *Fig use ~ Flower Availability* |
| Four | *Fig use ~ Fig Availability* |
| Five | *Fig use ~ Maximum mean temperature* |
| Six | *Fig use ~ Minimum mean temperature* |
| Seven | *Fig use ~ Rainfall* |
| Eight | *Fig use ~ Fruit Availability + Maximum mean temperature* |
| Nine | *Fig use ~ Fruit Availability + Maximum mean temperature+ Minimum mean temperature + Rainfall* |
| Ten | *Fig use ~ Seed Availability + Maximum mean temperature+ Minimum mean temperature + Rainfall* |
| Eleven | *Fig use ~ Flower Availability + Maximum mean temperature+ Minimum mean temperature + Rainfall* |
| Twelve | *Fig use ~ Fig Availability + Maximum mean temperature+ Minimum mean temperature + Rainfall* |

**Results of Model Selection for Fig Use**

| **Model** | **deltaAIC df Model weight** |
| --- | --- |

| One | 0 | 3 | 0.2306 |
| --- | --- | --- | --- |
| Two | 0 | 3 | 0.2251 |
| Three | 0.2 | 3 | 0.2078 |
| Four | 1.5 | 3 | 0.1089 |
| Zero | 1.7 | 2 | 0.0982 |
| Eight | 3.2 | 4 | 0.0474 |
| Six | 4.3 | 3 | 0.0272 |
| Seven | 4.4 | 3 | 0.0253 |
| Five | 4.5 | 3 | 0.0243 |
| Eleven | 10.2 | 6 | 0.0014 |
| Ten | 10.2 | 6 | 0.0014 |
| Nine | 10.5 | 6 | 0.0012 |
| Twelve | 11.2 | 6 | <0.001 |

**Leaf Monkeys and Leaf Use**

| **Model** | ***Structure*** |
| --- | --- |

| Zero | *Leaf use ~ Intercept* |
| --- | --- |
| One | *Leaf use ~ Fruit Availability* |
| Two | *Leaf use ~ Seed Availability* |
| Three | *Leaf use ~ Flower Availability* |
| Four | *Leaf Use ~ Fig Availability* |
| Five | *Leaf Use ~ Maximum mean temperature* |
| Six | *Leaf Use ~ Minimum mean temperature* |
| Seven | *Leaf Use ~ Rainfall* |
| Eight | *Leaf Use ~ Fruit Availability + Maximum mean temperature* |
| Nine | *Leaf Use ~ Fruit Availability + Maximum mean temperature+ Minimum mean temperature + Rainfall* |
| Ten | *Leaf Use ~ Seed Availability + Maximum mean temperature+ Minimum mean temperature + Rainfall* |
| Eleven | *Leaf Use ~ Flower Availability + Maximum mean temperature+ Minimum mean temperature + Rainfall* |
| Twelve | *Leaf Use ~ Fig Availability + Maximum mean temperature+ Minimum mean temperature + Rainfall* |

**Results of Model Selection for Leaf Use**

| **Model** | **deltaAIC df Model weight** |
| --- | --- |

| One | 0 | 3 | 0.3203 |
| --- | --- | --- | --- |
| Eight | 1.4 | 4 | 0.1607 |
| Three | 1.7 | 3 | 0.1353 |
| Zero | 2.2 | 2 | 0.1055 |
| Two | 2.8 | 3 | 0.0801 |
| Four | 3 | 3 | 0.0727 |
| Five | 3.7 | 3 | 0.051 |
| Seven | 4.4 | 3 | 0.0349 |
| Six | 4.7 | 3 | 0.0312 |
| Nine | 8.6 | 6 | 0.0044 |
| Twelve | 10.5 | 6 | 0.0017 |
| Eleven | 10.5 | 6 | 0.0016 |
| Ten | 12.3 | 6 | <0.001 |

**Leaf Monkeys and Seed Use**

| **Model** | ***Structure*** |
| --- | --- |

| Zero | *Seed use ~ Intercept* |
| --- | --- |
| One | *Seed use ~ Fruit Availability* |
| Two | *Seed use ~ Seed Availability* |
| Three | *Seed use ~ Flower Availability* |
| Four | *Seed use ~ Fig Availability* |
| Five | *Seed use ~ Maximum mean temperature* |
| Six | *Seed use ~ Minimum mean temperature* |
| Seven | *Seed use ~ Rainfall* |
| Eight | *Seed use ~ Fruit Availability + Maximum mean temperature* |
| Nine | *Seed use ~ Fruit Availability + Maximum mean temperature+ Minimum mean temperature + Rainfall* |
| Ten | *Seed use ~ Seed Availability + Maximum mean temperature+ Minimum mean temperature + Rainfall* |
| Eleven | *Seed use ~ Flower Availability + Maximum mean temperature+ Minimum mean temperature + Rainfall* |
| Twelve | *Seed use ~ Fig Availability + Maximum mean temperature+ Minimum mean temperature + Rainfall* |

**Results of Model Selection for Seed Use**

| **Model** | **deltaAIC df Model weight** |
| --- | --- |

| One | 0 | 3 | 0.3471 |
| --- | --- | --- | --- |
| Four | 2 | 3 | 0.1288 |
| Zero | 2.1 | 2 | 0.1244 |
| Three | 2.1 | 3 | 0.1185 |
| Eight | 2.6 | 4 | 0.0967 |
| Two | 3.1 | 3 | 0.0753 |
| Five | 4.4 | 3 | 0.0387 |
| Six | 4.6 | 3 | 0.0346 |
| Seven | 4.8 | 3 | 0.0308 |
| Nine | 10.3 | 6 | 0.002 |
| Twelve | 10.7 | 6 | 0.0017 |
| Eleven | 11.8 | 6 | <0.001 |
| Ten | 13.2 | 6 | <0.001 |

**Leaf Monkeys and Flower use**

| **Model** | ***Structure*** |
| --- | --- |

| Zero | *Flower use ~ Intercept* |
| --- | --- |
| One | *Flower use ~ Fruit Availability* |
| Two | *Flower use ~ Seed Availability* |
| Three | *Flower use ~ Flower Availability* |
| Four | *Flower use ~ Fig Availability* |
| Five | *Flower use ~ Maximum mean temperature* |
| Six | *Flower use ~ Minimum mean temperature* |
| Seven | *Flower use ~ Rainfall* |
| Eight | *Flower use ~ Fruit Availability + Maximum mean temperature* |
| Nine | *Flower use ~ Fruit Availability + Maximum mean temperature+ Minimum mean temperature + Rainfall* |
| Ten | *Flower use ~ Seed Availability + Maximum mean temperature+ Minimum mean temperature + Rainfall* |
| Eleven | *Flower use ~ Flower Availability + Maximum mean temperature+ Minimum mean temperature + Rainfall* |
| Twelve | *Flower use ~ Fig Availability + Maximum mean temperature+ Minimum mean temperature + Rainfall* |

**Results of Model Selection for Flower use**

| **Model** | **deltaAIC df Model weight** |
| --- | --- |

| Seven | 0 | 3 | 0.272 |
| --- | --- | --- | --- |
| Zero | 0.4 | 2 | 0.2209 |
| One | 1.4 | 3 | 0.1353 |
| Five | 2.2 | 3 | 0.0885 |
| Two | 3.1 | 3 | 0.0578 |
| Six | 3.1 | 3 | 0.0577 |
| Three | 3.2 | 3 | 0.0563 |
| Four | 3.2 | 3 | 0.0552 |
| Eight | 3.5 | 4 | 0.0467 |
| Nine | 9.1 | 6 | 0.0028 |
| Eleven | 9.4 | 6 | 0.0025 |
| Twelve | 9.5 | 6 | 0.0024 |
| Ten | 9.9 | 6 | 0.002 |

**Leaf Monkeys and Fruit use**

| **Model** | ***Structure*** |
| --- | --- |

| Zero | *Fruit use ~ Intercept* |
| --- | --- |
| One | *Fruit use ~ Fruit Availability* |
| Two | *Fruit use ~ Seed Availability* |
| Three | *Fruit use ~ Flower Availability* |
| Four | *Fruit use ~ Fig Availability* |
| Five | *Fruit use ~ Maximum mean temperature* |
| Six | *Fruit use ~ Minimum mean temperature* |
| Seven | *Fruit use ~ Rainfall* |
| Eight | *Fruit use ~ Fruit Availability + Maximum mean temperature* |
| Nine | *Fruit use ~ Fruit Availability + Maximum mean temperature+ Minimum mean temperature + Rainfall* |
| Ten | *Fruit use ~ Seed Availability + Maximum mean temperature+ Minimum mean temperature + Rainfall* |
| Eleven | *Fruit use ~ Flower Availability + Maximum mean temperature+ Minimum mean temperature + Rainfall* |
| Twelve | *Fruit use ~ Fig Availability + Maximum mean temperature+ Minimum mean temperature + Rainfall* |

**Results of Model Selection for Fruit use**

| **Model** | **deltaAIC df Model weight** |
| --- | --- |

| One | 0 | 3 | 0.474 |
| --- | --- | --- | --- |
| Two | 2.2 | 3 | 0.16 |
| Eight | 3.1 | 4 | 0.1 |
| Three | 3.4 | 3 | 0.086 |
| Zero | 3.6 | 2 | 0.079 |
| Four | 5.2 | 3 | 0.036 |
| Six | 6.2 | 3 | 0.021 |
| Five | 6.3 | 3 | 0.02 |
| Seven | 6.4 | 3 | 0.02 |
| Nine | 10.1 | 6 | 0.003 |
| Ten | 12.6 | 6 | <0.001 |
| Eleven | 13.8 | 6 | <0.001 |
| Twelve | 15.4 | 6 | <0.001 |
